# The freedom to explore: examining the influence of independent mobility on weekday, weekend and after-school physical activity behaviour in children living in urban and inner-suburban neighbourhoods of varying socioeconomic status

**DOI:** 10.1186/1479-5868-11-5

**Published:** 2014-01-22

**Authors:** Michelle R Stone, Guy EJ Faulkner, Raktim Mitra, Ron N Buliung

**Affiliations:** 1 School of Health and Human Performance, Faculty of Health Professions, Dalhousie University, 6230 South Street, PO Box 15000 Halifax, Nova Scotia B3H4R2, Canada; 2 Faculty of Kinesiology and Physical Education, University of Toronto, Toronto, Ontario, Canada; 3 School of Urban and Regional Planning, Ryerson University, Toronto, Ontario, Canada; 4 Department of Geography, University of Toronto Mississauga, Mississauga, Ontario, Canada

**Keywords:** Children’s independent mobility, Physical activity, Accelerometer, ActiGraph, Built environment, Child, Health

## Abstract

**Background:**

Children’s independent mobility (CIM) is critical to healthy development in childhood. The physical layout and social characteristics of neighbourhoods can impact opportunities for CIM. While global evidence is mounting on CIM, to the authors’ knowledge, Canadian data on CIM and related health outcomes (i.e., physical activity (PA) behaviour) are missing. The purpose of this study was to examine if CIM is related to multiple characteristics of accelerometry-measured PA behaviour (total PA, light PA, moderate-to-vigorous PA, time spent sedentary) and whether associations between CIM and PA behaviour systematically vary by place of residence, stratifying by gender and type of day/period (weekdays, after-school, weekend).

**Methods:**

Participants were recruited through Project BEAT (Built Environment and Active Transport; http://www.beat.utoronto.ca). Children (*n* = 856) were stratified into four neighbourhood classifications based on the period of neighbourhood development (urban built environment (BE) (old BE) versus inner-suburban BE (new BE)) and socioeconomic status (SES; low SES and high SES). Physical activity was measured via accelerometry (ActiGraph GT1M). CIM was assessed via parental report and two categories were created (low CIM, *n* = 332; high CIM, *n* = 524). A series of two-factor ANOVAs were used to determine gender-specific differences in PA for weekdays, weekend days and the after-school period, according to level of CIM, across four neighbourhood classifications.

**Results:**

Children who were granted at least some independent mobility (high CIM) had more positive PA profiles across the school week, during the after-school period, and over the weekend; they were also less sedentary. The influence of CIM on PA behaviour was particularly salient during the after-school period. Associations of CIM with PA varied by gender, and also by neighbourhood classification. CIM seemed to matter more in urban neighbourhoods for boys and suburban neighbourhoods for girls.

**Conclusion:**

Our findings highlight the importance of independent mobility to multiple characteristics of children’s PA behaviour across the week. Furthermore, they emphasize that independent mobility-activity relationships need to be considered by gender and the type of neighbourhood independent mobility is offered in. Future work will focus on developing a predictive model of CIM that could be used to inform decision-making around alleviating barriers to CIM.

## Introduction

Regular physical activity (PA) in childhood is associated with many physical, physiological and mental health benefits [1]. Like other countries, the majority (93%) of Canadian children and youth are not achieving a level of PA necessary to promote and maintain good health [2]; moreover, they have been failing to do so for quite some time [3]. Recent international comparisons of PA data reveal that Canadian children achieve among the lowest levels of PA [4]. In light of this evidence, it is perhaps not surprising that Canada, like the U.S., is experiencing a childhood obesity health crisis, an issue which recently led to the Public Health Agency of Canada’s creation of a federal, provincial and territorial framework on curbing childhood obesity (http://www.phac-aspc.gc.ca/hp-ps/hl-mvs/framework-cadre/index-eng.php). A key policy priority is the provision of supportive environments: “*making social and physical environments where children live, learn and play more supportive of physical activity and healthy eating*”.

Unfortunately, many children in Canada continue to be bound by social and physical barriers to PA participation, barriers that restrict independent mobility. Children’s Independent Mobility (CIM) is defined as “the freedom of children to travel around their own neighbourhood or city without adult supervision” [5]. This mobility could be for the purposes of play or travel, within and beyond their neighbourhood, and to destinations such as school and leisure facilities or simply just outside the home. This ability to move around independently is critical to children’s healthy development; it influences cognitive development [6], it helps children build relationships [7,8] which impacts social capital [9], and it allows children to engage/form bonds with other children and the natural environment [10,11]. CIM also assists with the development of movement skills [12-14] and affects many other aspects of health. For example, CIM has been shown to have a significant positive effect on PA [15-18], which we know is protective against obesity and chronic illness risk factors. For children, being independently mobile also translates to more time spent on foot and less time in the car, which may have important implications for pollution and air quality, both of which put children at risk for heart and respiratory diseases [19].

Data from the United Kingdom and Germany indicate that CIM has been in decline since the 1970s [20,21]. Since Hillman and Adams’ landmark study into CIM, further evidence of a reduction in CIM has emerged in Australia and New Zealand [22], again in England [23] and New Zealand [24,25], and in other countries such as Sweden [26], Italy [6], Denmark [27] and Finland and other Scandinavian countries [28,29]. CIM data are also now starting to emerge in Japan, South Africa and Tanzania [30]. This documentation of patterns and trends in children’s CIM across countries is providing valuable insight into cross-cultural differences. To the authors’ knowledge, Canadian data on CIM and related health outcomes are missing. As such, we are unable to contribute to global evidence that is amassing around this important child and youth mobility and health issue.

The decline in CIM over time has motivated an appreciable amount of research into the main factors underlying this change. One is perceived threats to safety; some suggest it to be the most prominent barrier to children’s independent play [31]. Concerns over safety can be tied to both the built form (neighbourhood design) and social framework (neighbourhood social capital). The physical layout of communities can therefore promote or inhibit opportunities for independent mobility, and in turn, PA. A recent review, for example, identified walkability, traffic speed/volume, access/proximity to recreation facilities, land-use mix and residential density as the most supported environmental correlates of children’s PA [32]. The social characteristics specific to a neighbourhood may also impact parents’ attitudes towards CIM. Socioeconomic status (SES) can vary widely across neighbourhoods, and therefore mediate the relationship of CIM with characteristics of the built environment. For example, children from lower SES neighbourhoods encounter greater safety risks on the way to school [33]. Parents of children living in high-walkability, low-income neighborhoods may express the most concerns with active travel to school [34]. Children from low SES households also tend to have lower PA levels and engage in more sedentary activities [35-37]. Given these relationships, it seems appropriate to account for SES within geographic features when investigating children’s PA.

We recently examined the relationship between school neighbourhood type (urban vs. inner-suburban) and SES (low vs. high, based on median household income reported in the 2006 Population Census of Canada) and physical activity in elementary school children [38]. While children living in more affluent neighbourhoods had more positive PA profiles across the school week, over the weekend, the influence of the neighbourhood design (i.e., urban vs. suburban) was stronger (PA profiles were highest in urban, high SES neighbourhoods). Furthermore, SES seemed to be a much stronger predictor of PA behaviour in girls than in boys. It was postulated that this could have resulted from girls being granted less independent mobility than boys which could be amplified in less affluent neighbourhoods because of heightened parental concerns regarding personal safety. There is consistent evidence that girls are granted less independent mobility than boys [39-49]. Moreover, girls are less physically active than boys [2], are less likely to travel actively to/from school [50] and engage in less outdoor play than boys [51,52]. Perhaps it is not surprising then that girls are less likely to meet PA recommendations for health benefits [2].

While this study [38] has generated important lessons on the relationship of both neighbourhood form and SES with PA in children, and highlighted the significance of considering gender and the type of day (weekday vs. weekend) in interpreting relationships with activity, what is missing is an understanding of whether CIM is related to multiple aspects of children’s PA behaviour, and, whether the influence of CIM on PA depends on where children live. Given that gender-specific differences in independent mobility and aspects of PA behaviour exist, it seems appropriate to stratify by gender when exploring these avenues of research. Therefore, the purpose of this study is to examine, a) if CIM is related to multiple characteristics of accelerometry-measured PA behaviour (total PA and time spent sedentary and in light and moderate-to-vigorous PA) and b) whether the association between CIM and PA behaviour systematically varies by place of residence, stratifying by gender and type of day/period (weekdays, weekend, after-school period).

## Methods

### Experimental design

Project BEAT (Built Environment and Active Transport; http://www.beat.utoronto.ca) is a large scale, multidisciplinary and mixed method study examining how the built environment influences school travel modes and other physical activity behaviour of elementary school children in Toronto. Marked differences in the built environment are visible across Toronto (for a historical description of changes to Toronto’s built form, see [38]). In the older central city (pre-World War II [53]) grid-based street networks dominate; intersections are denser and blocks typically short and straight (higher building densities and mixed land use also prevail). In the newer, inner-suburbs, neighbourhood streets are more curvilinear, land uses are segregated, housing density is lower, and there is more open space compared to the older neighbourhoods. SES varies widely across these central city (older) and inner-suburban (newer) neighbourhoods.

From January 2010 to June 2011, all elementary/intermediate schools within the Toronto District School Board with Grade 5 and 6 students (*n* = 469; age 10 to 12 years) received an invitation to participate. A pool of interested schools was generated and 16 schools selected that varied with respect neighbourhood type and level of SES. Neighbourhood classifications were created using the child’s home address. Two neighbourhood classifications were created on the basis of the period of neighbourhood development: urban built environment (BE) (old BE) versus inner-suburban BE (new BE); two classifications of SES (low SES and high SES) were also created, according to the median household income reported in the 2006 Population Census of Canada [38]. Consent was obtained from participating school boards, individual schools, parents, and students. Ethics approval from the Toronto District School Board (TDSB) and University of Toronto Office of Research Ethics was granted. Student participation was voluntary.

A total of 1,027 parents/guardians gave consent for their children to participate (boys, *n* = 478; girls, *n* = 549). Height and weight measurements were taken and accelerometer-measured physical activity collected on a total of 1,001 children. Of those, 85.5% had at least three weekdays and one weekend day of valid data (*n* = 856; boys = 389; girls = 467). With the use of age- and gender-specific body mass index (BMI) cut-points provided by the International Obesity Task Force [54], participants were classified as normal weight, overweight or obese.

### Physical activity measurement

Children’s PA was objectively measured for seven days using accelerometry (ActiGraph GT1M; ActiGraph LLC, Pensacola, FL, US). A 5 s epoch (interval) was used to capture the rapid transitions in activity that are typical of children [55]. For inclusion in data analysis, each child required a minimum of 10 hours of wear time for at least 3 weekdays and 1 weekend day [56]. Children were asked to wear their accelerometer consistently and only remove the device when engaging in water-based activities. Time spent at various levels of movement intensity was classified according to published thresholds for children [57] and used to determine levels of PA during school days (weekdays), weekends and during the after-school period (2 hours immediately after the end-of-school bell). Physical activity variables of interest included total physical activity (counts^.^day^-1^), time spent sedentary (% of day) and minutes of light-intensity physical activity and moderate-to-vigorous physical activity (MVPA). Data collection took place during the spring/summer (April to June) and fall (September to December) school periods to limit any seasonal effect.

### Measurement of independent mobility

CIM was assessed via parental report. Parents were asked the following question: “In general, how often do you allow your child to go out on their own or with friends without an adult?” Parents then reported one of the following options: never; sometimes; often; always [58]. A frequency analysis was conducted and results used to establish two CIM categories: (1) never allowing the child out without adult accompaniment (i.e., low independent mobility [low CIM; *n* = 332]) and (2) sometimes, often or always allowing the child out without adult accompaniment (i.e., high independent mobility (high CIM; *n* = 524)).

### Statistical analyses

A series of multivariate ANOVAs were used to determine gender-specific differences in weekday, weekend and after-school characteristics of physical activity (total PA, light PA, MVPA) and inactivity (time spent sedentary) according to level of CIM (low CIM; high CIM). A series of two-factor ANOVAs were used to explore these same aspects of PA and inactivity by level of CIM and type of neighbourhood (old BE, low SES (OL); old BE, high SES (OH); new BE, low SES (NL); new BE, high SES (NH)). Gender-specific differences in descriptive characteristics (age, height, weight, BMI and proportion of normal weight and overweight/obese participants) were also explored according to level of CIM, and across the four neighbourhood classifications. Estimated means were compared and significant differences tested using the Sequential Bonferroni method. The alpha level was set at 0.05. SPSS version 20.0 was used for all analyses.

## Results

### Descriptive characteristics

Data for 856 participants are presented (mean age 11.0 ± 0.6 years; boys, *n* = 389, girls, *n* = 467, Table 1). In general, children were more likely to be granted some independent mobility than none at all (high CIM = 61.2% vs. low CIM = 39.8% of the sample). However, the level of independent mobility afforded to children varied according to gender, with a greater proportion of boys being granted high CIM than girls (69.4% vs. 54.4%, respectively). Descriptive analyses revealed that there were no significant differences in weight (kg) or BMI (kg^.^m^-2^) between groups (*p* > 0.05). However, boys who were allowed a greater degree of independent mobility were significantly taller and older in comparison to boys who were never allowed out without an adult (height: F_388_ = 10.2, *p* = 0.002; age: F_388_ = 6.8, *p* = 0.009, Table 2). When examined across neighbourhood classifications, there was a significant main effect of CIM (*F*_1,388_ = 11.6, *p* = 0.001) and neighbourhood classification (*F*_3,388_=, 5.9, *p* = 0.001) on age in boys, but no significant interaction (*F*_3,388_ = 0.8, *p* = 0.482, Table 2). Across all neighbourhoods, boys who were older were granted more CIM; boys in NH neighbourhoods were significantly older than boys in NL and OL neighbourhoods (NL: mean difference = 0.25 years, 95% CI, 0.06 to 0.43; OL: mean difference = 0.30 years, 95% CI, 0.02 to 0.58). No significant differences emerged in girls (Table 3). The differences in age and height between boys granted low and high independent mobility are relatively small and unlikely to be of practical significance; therefore, age and height were not controlled for in subsequent analyses.

**Table 1:** Descriptive characteristics and weekday, weekend and after-school physical activity of boys (n = 389) and girls (n = 467) by level of children’s independent mobility (CIM) (Toronto, Ontario, Canada; 2010–2011)

**Variable**				
	**Low CIM**	**High CIM**	**Low CIM**	**High CIM**
**Descriptive characteristics**	**Boys**		**Girls**	
Sample size	119	270	213	254
Age (years)	10.9 (0.6)	11.1 (0.6)^a^	11.0 (0.6)	11.1 (0.6)
Height (cm)	145.7 (7.8)	147.8 (7.2)^a^	146.8 (8.3)	147.9 (10.2)
Weight (kg)	41.3 (10.7)	42.7 (10.9)	40.6 (9.4)	41.1 (9.3)
Body mass index (kg^.^m^-2^)	19.2 (3.8)	19.4 (3.9)	18.7 (3.2)	18.5 (3.2)
BMI category‡				
Normal weight, %	61.3	70.0	73.1	74.4
Overweight or obese, %	38.7	30.0	26.9	25.6
**Weekday physical activity**				
Total counts (counts.day^-1^)	472530.2 (118847.7)	509174.8 (140120.3)^a^	379470.3 (102001.4)	406276.1 (110737.3)^a^
Light activity (min)	190.6 (33.2)	193.0 (33.4)	169.5 (30.1)	173.6 (32.1)
MVPA (min)	36.1 (14.6)	40.4 (16.1)^a^	24.9 (11.0)	27.5 (12.1)^a^
Time spent sedentary (%)	77.3 (4.9)	76.5 (5.4)	80.6 (4.2)	79.2 (4.8)^b^
**Weekend physical activity**				
Total counts (counts.day^-1^)	360493.0 (131742.8)	395607.5 (179477.2)^a^	287227.3 (109164.1)	341835.3 (172700.0)^a^
Light activity (min)	164.2 (40.3)	168.5 (50.0)^a^	143.0 (39.8)	151.0 (39.1)
MVPA (min)	24.2 (14.6)	27.5 (16.7)^a^	16.9 (10.7)	20.9 (15.2)
Time spent sedentary (%)	80.2 (5.4)	79.2 (7.1)	82.8 (5.6)	81.0 (6.0)
**After-school physical activity**				
% time sedentary	72.2 (8.9)	68.0 (9.5)^b^	74.6 (6.9)	72.4 (7.6)^b^
% time in light activity	23.7 (7.2)	26.2 (7.3)^a^	22.2 (5.6)	23.7 (6.4)^a^
% time in MVPA	4.2 (2.9)	5.9 (3.6)^a^	3.2 (2.2)	3.9 (2.5)^a^

Group differences explored using multivariate analyses (MANOVAs).

Mean (SD) presented.

High CIM = high children’s independent mobility; low CIM = low children’s independent mobility.

^a^Significantly higher in those children granted high CIM (*p* < 0.05).

^b^Significantly lower in those children granted high CIM (*p* < 0.05).

‡International Obesity Task Force Classification [54].

**Table 2:** Descriptive characteristics and weekday, weekend and after-school physical activity of boys (n = 389), according to level of children’s independent mobility (CIM) and neighbourhood classification (Toronto, Ontario, Canada; 2010–2011)

**Boys (*n*** **= 389)**	**Old built environment, low SES (*n*** **= 121)**		**Old built environment, high SES (*n*** **= 66)**		**New built environment, low SES (*n*** **= 37)**		**New built environment, high SES (*n*** **= 165)**	
**Descriptive characteristics**	**Low CIM**	**High CIM**	**Low CIM**	**High CIM**	**Low CIM**	**High CIM**	**Low CIM**	**High CIM**
Sample size	34	87	12	54	11	26	62	103
Age (years)^a,b^	10.8 (0.1)	11.0 (0.1)^c^	10.9 (0.2)	11.1 (0.1)^c^	10.5 (0.2)	11.0 (0.1)^c^	11.0 (0.1)	11.2 (0.1)^c^
Height (cm)	144.8 (1.2)	148.0 (0.8)	146.0 (2.1)	147.1 1.0)	144.3 (2.2)	145.7 (1.5)	146.4 (0.9)	148.7 (0.7)
Weight (kg)	42.0 (1.8)	42.7 (1.2)	38.9 (3.1)	39.9 1.5)	44.9 (3.3)	43.5 (2.1)	40.7 (1.4)	44.0 (1.1)
Body mass index (kg^.^m^-2^)^b^	19.7 (0.7)	19.3 (0.4)	18.1 (1.1)	18.3 (0.5)	21.4 (1.2)	20.3 (0.8)	18.8 (0.5)	19.8 (0.4)
BMI category‡								
Normal weight, %	58.8	71.3	75.0	77.8	27.3	57.7	66.1	68.0
Overweight or obese, %	41.2	28.7	25.0	22.2	72.7	42.3	33.9	32.0
**Weekday physical activity**								
Total counts (counts.day^-1^)^a, b^	446395 (22729)	489183 (14209)	432406 (38259)	512844 (18036)	430724 (39961)	469691 (25992)	502046 (16831)	534104 (13059)
Light activity (min)^b^	192.4 (5.7)	190.4 (3.5)	161.8 (9.5)^c^	192.0 (4.5)	184.8 (10.0)	191.2 (6.5)	196.2 (4.2)	196.1 (3.3)
MVPA (min)^b^	32.1 (2.7)^c^	38.7 (1.7)	35.6 (4.5)	39.1 (2.1)	29.5 (4.7)	35.7 (3.0)	39.5 (2.0)	43.7 (1.5)
Time spent sedentary (%)^b^	77.2 (0.9)	78.0 (0.6)	77.6 (1.5)	75.5 (0.7)	79.3 (1.6)	79.3 (1.0)	77.0 (0.6)^d^	75.1 (0.5)
**Weekend physical activity**								
Total counts (counts.day^-1^)^a^	312265 (28142)	372054 (17593)	334538 (47370)^c^	457934 (22330)	329524 (49476)	387017 (32181)	397459 (20839)	384995 (16169)
Light activity (min)^a^	157.8 (8.0)	160.8 (5.0)	147.6 (13.5)^c^	178.9 (6.4)	159.4 (14.1)	182.6 (9.2)	171.8 (6.0)	165.9 (4.6)
MVPA (min)^a,b^	18.6 (2.7)^c^	25.8 (1.7)	23.3 (4.6)	31.4 (2.2)	19.5 (4.8)	25.5 (3.1)	28.3 (1.7)	27.2 (1.6)
Time spent sedentary (%)^b^	80.8 (1.1)	81.1 (0.7)	77.9 (1.9)	76.5 (0.9)	83.0 (2.0)	80.8 (1.3)	79.6 (0.8)	78.6 (0.6)
**After-school physical activity**								
% time sedentary^a^	72.4 (1.6)^d^	67.0 (1.0)	77.0 (2.7)^d^	66.6 (1.3)	69.2 (2.8)	68.7 (1.8)	71.6 (1.2)	69.4 (0.9)
% time in light activity^a^	24.0 (1.2)^c^	26.9 (0.8)	19.0 (2.1)^c^	27.1 (1.0)	26.3 (2.2)	25.4 (1.4)	24.0 (0.9)	25.2 (0.7)
% time in MVPA^a^	3.6 (0.6)^c^	6.1 (0.4)	4.1 (1.0)^c^	6.3 (0.5)	4.5 (1.0)	5.9 (0.7)	4.4 (0.4)	5.4 (0.3)

Group differences explored using two-factor ANOVAs.

Mean (SE) presented.

High CIM = high children’s independent mobility; low CIM = low children’s independent mobility.

SES = socioeconomic status.

^a^CIM difference (main effect, p < 0.05).

^b^Neighbourhood difference (main effect, p < 0.05).

^c^CIM x neighbourhood interaction (p < 0.05); significantly lower in those boys granted low CIM (p < 0.05).

^d^CIM x neighbourhood interaction (p < 0.05); significantly higher in those boys granted low CIM (*p* < 0.05).

‡International Obesity Task Force Classification.

**Table 3:** Descriptive characteristics and weekday, weekend and after-school physical activity of girls (n = 467), according to level of children’s independent mobility (CIM) and neighbourhood classification (Toronto, Ontario, Canada; 2010–2011)

**Girls (*n*** **= 467)**	**Old built environment, low SES (*n*** **= 135)**		**Old built environment, high SES (*n*** **= 89)**		**New built environment, low SES (*n*** **= 67)**		**New built environment, high SES (*n*** **= 175)**	
**Descriptive characteristics**	**Low CIM**	**High CIM**	**Low CIM**	**High CIM**	**Low CIM**	**High CIM**	**Low CIM**	**High CIM**
Sample size	57	78	18	71	31	36	106	69
Age (years)	10.9 (0.1)	11.1 (0.1)	10.8 (0.1)	11.2 (0.1)	11.0 (0.1)	10.9 (0.1)	11.1 (0.1)	11.2 (0.1)
Height (cm)	146.3 (1.2)	147.2 (1.1)	148.3 (2.2)	150.3 (1.1)	145.6 (1.7)	147.5 (1.6)	147.2 (0.9)	146.6 (1.1)
Weight (kg)	39.4 (1.2)	40.6 (1.1)	39.3 (2.2)	40.4 (1.1)	41.4 (1.7)	43.1 (1.6)	41.2 (0.9)	41.2 (1.1)
Body mass index (kg^.^m^-2^)	18.3 (0.4)	18.6 (0.4)	17.7 (0.8)	17.8 (0.4)	19.4 (0.6)	19.6 (0.5)	18.8 (0.3)	18.7 (0.4)
BMI category‡								
Normal weight, %	84.2	75.6	83.3	83.1	61.3	58.3	68.9	72.5
Overweight or obese, %	15.8	24.4	16.7	16.9	38.7	41.7	31.1	27.5
**Weekday physical activity**								
Total counts (counts.day^-1^)^a^	387241 (14183)	408281 (12125)	412248 (25239)	407490 (12708)	353042 (19232)	401537 (17847)	377455 (10401)	405234 (12891)
Light activity (min)^b^	175.3 (4.1)	180.1 (3.5)	165.0 (7.3)	164.4 (3.7)	171.3 (5.6)	177.8 (5.2)	166.6 (3.0)	173.5 (3.7)
MVPA (min)^b^	25.1 (1.5)	26.9 (1.3)	31.2 (2.7)	29.2 (1.4)	20.8 (2.1)^c^	26.8 (1.9)	24.9 (1.1)	26.7 (1.4)
Time spent sedentary (%)^a,b^	80.9 (0.6)	79.6 (0.5)	79.6 (1.1)	78.1 (0.5)	82.3 (0.8)	80.5 (0.7)	80.0 (0.4)	79.2 (0.5)
**Weekend physical activity**								
Total counts (counts.day^-1^)^a^	287499 (19398)	327949 (16582)	343401 (34519)	375982 (17381)	310993 (26304)	310754 (24409)	270592 (14225)^c^	338612 (17631)
Light activity (min)^b^	146.8 (5.1)	152.9 (4.4)	155.4 (9.2)	155.1 (4.6)	161.0 (7.0)	155.4 (6.5)	133.5 (3.8)	142.5 (4.7)
MVPA (min)^b^	16.2 (1.7)	18.3 (1.5)	23.1 (3.1)	26.8 (1.6)	17.2 (2.3)	17.7 (2.2)	16.2 (1.3)	19.6 (1.6)
Time spent sedentary (%)^b^	83.5 (0.7)	81.9 (0.6)	80.6 (1.3)	78.3 (0.7)	82.0 (1.0)	82.5 (0.9)	83.0 (0.6)	82.2 (0.7)
**After-school physical activity**								
% time sedentary^a,b^	74.1 (1.0)^d^	70.8 (0.8)	73.4 (1.7)	73.0 (0.9)	74.0 (1.3)	71.8 (1.2)	75.3 (0.7)	74.1 (0.9)
% time in light activity ^a,b^	22.9 (0.8)^c^	25.3 (0.7)	22.7 (1.4)	22.8 (0.7)	23.2 (1.1)	24.2 (1.0)	21.5 (0.6)	22.4 (0.7)
% time in MVPA^a^	3.1 (0.3)	3.9 (0.3)	3.9 (0.6)	4.2 (0.3)	2.8 (0.4)^c^	4.1 (0.4)	3.2 (0.2)	3.5 (0.3)

Group differences explored using two-factor ANOVAs.

Mean (SE) presented.

*High CIM* = high children’s independent mobility; low CIM = low children’s independent mobility.

*SES* socioeconomic status.

^a^CIM difference (main effect, p < 0.05).

^b^Neighbourhood difference (main effect, p < 0.05).

^c^CIM x neighbourhood interaction (p < 0.05); significantly lower in those girls offered low CIM (p < 0.05).

^d^CIM x neighbourhood interaction (p < 0.05); significantly higher in those girls granted low CIM (*p* < 0.05).

‡International Obesity Task Force Classification.

### Weekday physical activity

Children granted a higher degree of independent mobility (high CIM) accumulated significantly more total weekday physical activity (boys: F_388_ = 6.2, *p* = 0.013; girls: F_467_ = 7.3, *p* = 0.017) and moderate-to-vigorous physical activity (boys: F_388_ = 6.2, *p* = 0.013; girls: F_467_ = 5.8, *p* = 0.017) in comparison to children who were never allowed out without an adult present (low CIM) (Table 1). Time spent sedentary was also lower in girls (but not boys) who were granted high CIM compared to their low CIM counterparts (F_467_ = 10.5, *p* = 0.001). The accumulation of light physical activity on weekdays was similar between those granted low and high CIM (*p* > 0.05, Table 1).

When the impact of CIM on weekday PA was examined across neighbourhood classifications, significant interactions emerged. Boys who were granted low CIM in OL and OH neighbourhoods accumulated less MVPA and light physical activity, respectively, across the school week compared to those offered greater licence to explore their neighbourhood without adult supervision (OL: mean difference in MVPA = 7.0 minutes, 95% CI, 0.46 to 12.8; OH: mean difference in light PA = 30.2 minutes, 95% CI, 9.5 to 50.1). In NH neighbourhoods, restrictions on boys’ independent mobility corresponded with a greater proportion of the day spent sedentary (mean difference = 1.8%, 95% CI, 0.21 to 3.5, Table 2). For girls, the accumulation of MVPA across the school week was significantly lower amongst those granted low CIM in suburban, low SES neighbourhoods (MVPA: mean difference = 6.1 minutes, 95% CI, 0.50 to 11.6, Table 3).

### Weekend physical activity

Similar to weekday data, children who were granted greater independent mobility over the weekend had more positive physical activity profiles than those whose independent mobility was restricted (Table 1). Boys granted high CIM accumulated significantly more total PA (F_388_ = 3.7, *p* = 0.040) and MVPA (F_388_ = 3.4, *p* = 0.049) than boys with low CIM. All aspects of weekend physical activity (total, light and MVPA) were higher, and time spent sedentary lower, amongst girls granted high CIM (*p* < 0.05, Table 1).

When the impact of CIM on weekend PA was examined across neighbourhood classifications, again, significant interactions emerged (Tables 2 and 3). Boys who were granted low CIM in OL neighbourhoods accumulated less MVPA (mean difference = 7.3 minutes, 95% CI, 0.94 to 13.6) and those in OH neighbourhoods less total physical activity and light physical activity across the weekend (total PA: mean difference = 123397 counts^.^day^-1^, 95% CI, 20428 to 226366; light physical activity: mean difference = 31.3 minutes, 95% CI, 1.9 to 60.7, Table 2). Alternatively, those girls in suburban, high SES neighbourhoods whose CIM was restricted accumulated significantly less total physical activity on the weekend compared to those offered more licence (mean difference = 68020 counts^.^day^-1^, 95% CI = 23502 to 112538, Table 3).

### After-school activity

Similar to weekday and weekend physical activity data, those children who were granted greater CIM were significantly more active during the two hours directly following the end-of-school day (*p* < 0.05, Table 1). When the impact of CIM on after-school PA was examined across neighbourhood classifications, significant interactions emerged. Boys who were granted low CIM in OL and OH neighbourhoods spent a significantly greater proportion of the after-school period sedentary than boys granted high CIM (OL: mean difference = 5.3%, 95% CI, 1.6 to 9.0; OH: mean difference = 10.4%, 95% CI, 4.6 to 16.2%), and also spent less of this time accumulating light and moderate-to-vigorous intensity PA (% time spent in light activity: OL: mean difference = 2.9%, 95% CI, 0.02 to 5.8; OH: mean difference = 8.2%, 95% CI, 3.6 to 12.7%; % time in MVPA: OL: mean difference = 2.4%, 95% CI, 1.1 to 3.8; OH: mean difference = 2.2%, 95% CI, 0.08 to 4.4) (Table 2).

Like boys, girls in OL neighbourhoods who were granted low CIM also spent more of the after-school period sedentary and less of this time accumulating light intensity activity (% time spent sedentary: mean difference = 3.2%, 95% CI, 0.78 to 5.7; % time in light activity: mean difference = 2.4%, 95% CI, 0.37 to 4.5). Furthermore, girls in suburban, low SES neighbourhoods whose CIM was restricted spent less of the after-school period accumulating MVPA than girls who were granted more independent licence in these neighbourhoods (mean difference = 1.3%, 95% CI, 0.10 to 2.4) (Table 3).

## Discussion

This study investigated whether characteristics of accelerometry-measured physical activity behaviour across the school week and over the weekend vary according to the amount of independent mobility a child is granted, and, whether the type of neighbourhood in which a child resides in (urban vs. inner-suburban, low vs. high SES) is associated with any observed relationships. We found that over half (61%) of children were offered some sort of independent mobility (i.e., were sometimes, often or always allowed out without adult accompaniment); the likelihood of being granted some independent mobility, however, differed according to the child’s gender and age. Nearly 70% of boys were offered this independent licence, compared to just over half (54%) of girls. Age appeared to be a moderating factor, but only in boys, with older (and in fact taller) boys being granted more independent mobility than younger, shorter boys. These differences in age and height were however relatively small (likely a result of the narrow age range of the sample (10 to 12 years)) and of not enough practical significance to justify controlling for in further analyses. Nevertheless, our findings support previous studies suggesting that age is associated with CIM [31] and also that gender is a strong correlate, with boys experiencing more independent mobility than girls [39-49].

Children who were granted at least some independent mobility had more positive physical activity profiles across the school week, over the weekend, and during the after-school period. Importantly, all characteristics of physical activity behaviour (total physical activity, and time spent in light and MVPA) were significantly greater, and time spent sedentary significantly lower, in comparison to children whose IM was restricted. Our findings are in line with previous evidence showing positive associations between independent mobility and children’s physical activity [15-18]. Our findings contribute to the CIM and health literature by examining possible associations with other aspects of physical activity behaviour in children (i.e., sedentary behaviour [59], light intensity activity, total physical activity), particularly during discrete periods of the week (weekdays, after-school period, weekend). The fact that this is the first study to identify associations between CIM and sedentary behaviour and light physical activity in particular is noteworthy, given a) increasing evidence that sedentary behaviour, in comparison to physical activity, has very different, independent, negative effects on human metabolism, physical function and health outcomes [60-68] and, b) increasing priority towards shifting time spent sedentary to time spent in light physical activity (i.e. minimizing sedentary behaviour and maximizing light physical activity) [69]. For example, more recent evidence suggests that health benefits accrue when sedentary time is replaced by light physical activity [63]; including measures of light physical activity in explorations with health outcomes is now strongly recommended [70].

While a greater degree of CIM is related to a more optimal physical activity profile in general, gender-specific relationships are apparent. For example, the level of CIM granted to girls impacts all characteristics of PA, with the exception of light PA accumulated across the school week. Like girls, CIM does not appear to have an impact on boys’ accumulation of light PA on weekdays, nor does it seem to impact light PA accumulated over the weekend. Time spent sedentary is also no different between boys granted high or low CIM. Interestingly all characteristics of PA during the after-school period are affected by the level of independent mobility afforded to boys and girls, suggesting that the influence of IM on PA behaviour is particularly salient during this time period. The overall results suggest that CIM-PA relationships are different for boys and girls, with certain aspects of “periodic activity” (i.e., after-school period) impacted more than others. Ultimately, this emphasizes the importance of considering gender and the type of day/period in future examinations of CIM-PA relationships.

When the impact of independent mobility on characteristics of physical activity behaviour is examined across neighbourhood classification, significant interactions emerge. However, similarly, these interactions vary according to gender, and the type of day (weekday, weekend) or period (after-school) being examined. Independent mobility seems to matter more for boys who live in urban neighbourhoods and for girls who live in suburban neighbourhoods. For example, boys offered greater independent mobility in urban neighbourhoods, of both low and high SES, have more positive physical activity profiles (i.e., accumulate more PA and spend less time sedentary) across the week and during the after-school period than boys whose licence is restricted in these neighbourhoods. Alternatively, girls in suburban, low SES neighbourhoods whose independent mobility is not restricted have more positive weekday and after-school physical activity profiles than girls who face restrictions in these neighbourhoods. On the weekend, however, CIM appears to have the strongest relationship with the physical activity patterns of girls in suburban, high SES neighbourhoods (i.e., the difference in weekend PA between girls granted low or high CIM is greatest in this neighbourhood).

The influence of CIM in these neighbourhoods also impacts different aspects of physical activity behaviour. In NL neighbourhoods, CIM seems to have the greatest impact on girls’ accumulation of MVPA across the school week and during the after-school period, whereas in NH neighbourhoods, total physical activity is impacted most on at the weekend. Restricting girls’ independent mobility in OL neighbourhoods seems to be related to more time spent sedentary, and less time in light activity, during the after-school period. For boys, restricting independent mobility in OL neighbourhoods is related to a lower accumulation of MVPA, across the school week and over the weekend, whereas light intensity (and total PA on the weekend) is impacted most in OH neighbourhoods. The after-school period, however, seems to be most affected in these urban neighbourhoods: no CIM is associated with more time spent sedentary and less time in light and MVPA. The after-school period is an opportune time to accumulate physical activity, however, for most Canadian children it remains underutilized. Our results show that children are spending anywhere from 68-75% of the after-school period sedentary (up to an hour and a half of those two hours sedentary), and only 3-6% of that time in MVPA (4 to 7 minutes). A better understanding of barriers towards CIM, particularly during this critical period, is a necessary step towards shifting these proportions.

### Strengths and limitations

Strengths of this study include the large sample (*n* = 856), the sampling methodology (stratification of children according to urban vs. inner-suburban, low vs. high SES neighbourhoods, using home address data) and the use of an objective measure of physical activity to examine numerous characteristics of physical activity behaviour during weekdays, the after-school period, and over the weekend. The investigation of the entire physical activity intensity spectrum (i.e., not only time spent in MVPA during these periods, but also time spent sedentary and in light activity) supports increasing evidence around the importance of assessing time spent sedentary and in light and MVPA, given independent relationships with various health outcomes exist. The collection of high-frequency physical activity data was also appropriate for describing children’s physical activity behaviour [55]. The limitations of this study include the narrow age range of children sampled and the examination of children living in neighbourhoods throughout the city of Toronto, preventing the generalizability of findings to other age groups and locations. Also, there is the possibility of inflated type I error due to multiple statistical comparisons. Finally, micro-level community design and land-use characteristics (e.g. connectivity, access/proximity to recreational facilities, residential density) were not examined in relation to both CIM and PA behaviour; these are a focus of future investigation.

## Conclusion

In conclusion, our findings highlight the importance of independent mobility to multiple characteristics of children’s physical activity behaviour (total activity, time spent sedentary and in light and MVPA) across the entire week (i.e., across weekdays, during the after-school period and over the weekend). Moreover, our work offers up three important lessons: one, that age and gender are associated with the amount of independent mobility afforded to a child; two, that being offered at least some independent mobility is related to more positive physical activity profiles; three, that independent mobility-activity relationships need to be considered by gender and the type of neighbourhood independent mobility is offered in (boys = urban; girls = suburban). Our findings have now provided a basis for some more in-depth investigations into specific correlates of CIM, using micro-level built form data, traffic data and information collected through our parental/child questionnaires, to develop a predictive model of CIM. This model will ultimately be used to inform decision-making around alleviating barriers to children’s independent mobility.

## Competing interests

The authors declare that they have no competing interests.

## Authors’ contributions

MS coordinated the study, conducted data collection, performed data analyses, and drafted the manuscript. GF and RB conceived of the study. GF, RB and RM participated in its design and coordination and helped to draft the manuscript. All authors read and approved the final manuscript.

## Authors’ information

MS is an Assistant Professor in the School of Health and Human Performance at Dalhousie University. Her interests are concerned with physical activity measurement, in particular, exploring relationships between the pattern of physical activity/inactivity and health outcomes in children, and, examining the role of the environment on children’s physical activity behaviour and health.

GF is a Professor in the Faculty of Kinesiology and Physical Education at the University of Toronto. His work is concerned with physical activity and public health.

RM is an Assistant Professor in the School of Urban and Regional Planning at Ryerson University. His work is concerned with land-use transportation planning and healthy communities planning.

RB is an Associate Professor in the Department of Geography at the University of Toronto Mississauga. His research interests include child and youth mobility, citizenship, and health.
